# An Intrinsic Alkalization Circuit Turns on *mntP* Riboswitch under Manganese Stress in Escherichia coli

**DOI:** 10.1128/spectrum.03368-22

**Published:** 2022-10-03

**Authors:** Arunima Kalita, Rajesh Kumar Mishra, Vineet Kumar, Amit Arora, Dipak Dutta

**Affiliations:** a CSIR Institute of Microbial Technology, Chandigarh, India; University of Minnesota

**Keywords:** manganese toxicity, *mntP* riboswitch, intrinsic alkalization, pH elevation, glutamine synthetase, glutamine, glutaminases, ammonia production, *Escherichia coli*

## Abstract

The trace metal manganese in excess affects iron-sulfur cluster and heme-protein biogenesis, eliciting cellular toxicity. The manganese efflux protein MntP is crucial to evading manganese toxicity in bacteria. Recently, two Mn-sensing riboswitches upstream of *mntP* and *alx* in Escherichia coli have been reported to mediate the upregulation of their expression under manganese shock. As the *alx* riboswitch is also responsive to alkaline shock administered externally, it is intriguing whether the *mntP* riboswitch is also responsive to alkaline stress. Furthermore, how both manganese and alkaline pH simultaneously regulate these two riboswitches under physiological conditions is a puzzle. Using multiple approaches, we show that manganese shock activated glutamine synthetase (GlnA) and glutaminases (GlsA and GlsB) to spike ammonia production in E. coli. The elevated ammonia intrinsically alkalizes the cytoplasm. We establish that this alkalization under manganese stress is crucial for attaining the highest degree of riboswitch activation. Additional studies showed that alkaline pH promotes a 17- to 22-fold tighter interaction between manganese and the *mntP* riboswitch element. Our study uncovers a physiological linkage between manganese efflux and pH homeostasis that mediates enhanced manganese tolerance.

**IMPORTANCE** Riboswitch RNAs are *cis*-acting elements that can adopt alternative conformations in the presence or absence of a specific ligand(s) to modulate transcription termination or translation initiation processes. In the present work, we show that manganese and alkaline pH are both necessary for maximal *mntP* riboswitch activation to mitigate the manganese toxicity. This study bridges the gap between earlier studies that separately emphasize the importance of alkaline pH and manganese in activating the riboswitches belonging to the *yybP*-*ykoY* family. This study also ascribes a physiological relevance as to how manganese can rewire cellular physiology to render cytoplasmic pH alkaline for its homeostasis.

## INTRODUCTION

Manganese is a crucial metal that determines the pathogenic potential of bacteria (1–3). This metal has been shown to sustain the functions of a small subset of proteins, such as superoxide dismutase and ribonucleotide reductase, under iron starvation and oxidative stress (4–6). Conversely, iron starvation and oxidative stress are also induced in Escherichia coli in a laboratory setting by the presence of excess manganese in the medium (7–9). Excess manganese at a toxic level impairs the biogenesis of iron-sulfur cluster and heme-containing proteins in E. coli, thereby compromising the function of proteins involved in the electron transport chain, affecting energy metabolism (8–10).

A manganese-dependent transcription regulator (MntR) downregulates the manganese importer (*mntH*) and upregulates the manganese exporter (*mntP*) to alleviate manganese toxicity in E. coli (7). Notably, MntP-mediated efflux of manganese alone efficiently controls the detrimental effects of manganese overload (7–9). As a result, a Δ*mntP* strain is more sensitive to manganese than wild-type (WT) E. coli cells (7). Interestingly, a recent study has demonstrated that a 5′-untranslated region (5′-UTR) of *mntP* transcript forms a manganese-dependent riboswitch to turn on translation initiation of *mntP*, causing successful manganese efflux (11).

Riboswitch RNAs can adopt at least two alternative conformations depending on specific ligand(s) availability to modulate transcription termination or translation initiation processes (12). The *mntP* riboswitch and another riboswitch at the 5′-UTR of the *alx* gene in E. coli belong to the *yybP-ykoY* riboswitch family that has been shown to be regulated by manganese (11, 13–15). However, the preceding reports suggest that the *alx* riboswitch is also responsive to alkaline shock administered externally (11, 16, 17). In this context, it is intriguing whether the *mntP* riboswitch also responds to alkaline stress.

We previously demonstrated that manganese stress inhibits the activity of glutamate synthase (GOGAT), an iron-dependent enzyme (9). Together with this information, our present work reveals that manganese shock intrinsically spikes cellular ammonia levels by altering the activities of the glutamate-glutamine cycle enzymes, *viz.*, GOGAT, glutamine synthetase (GS) (GlnA), and glutaminase (Gls) (GlsA and GlsB) enzymes. This ammonia production elevates cellular pH, favoring the activation of the *mntP* riboswitch.

## RESULTS

### Glutamine synthetase function is critical for manganese homeostasis.

**FIG 1:**
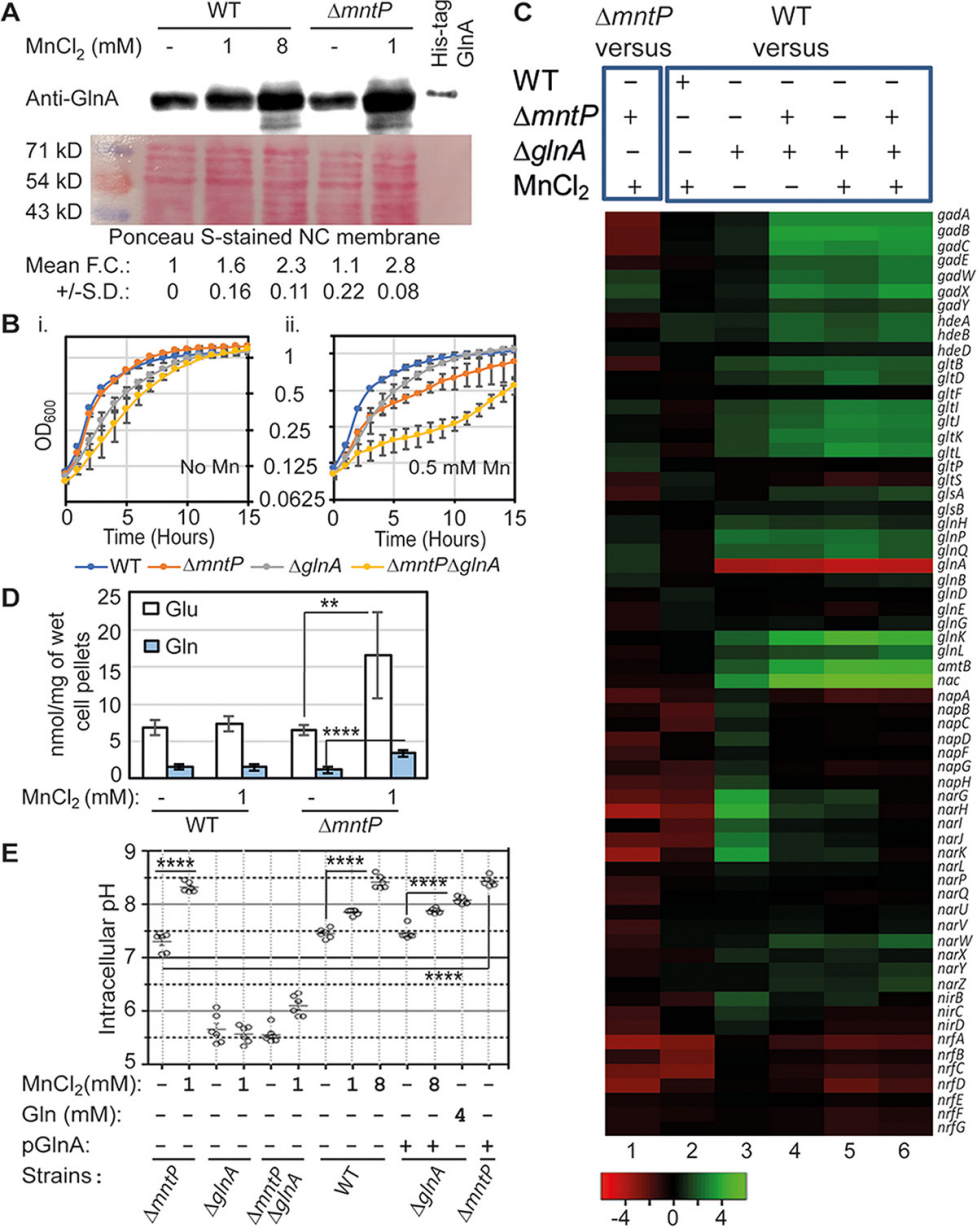
Manganese-mediated GlnA activation triggers intracellular pH elevation. (A) Western blot shows upregulated GlnA in the manganese-fed WT and Δ*mntP* strains. The corresponding ponceau-stained nitrocellulose membrane was represented to show equal loading of the cellular proteins. The fold change (FC) values are the mean ± SD from three independent blots. (B) Growth curves show that the Δ*mntP* Δ*glnA* double mutant is extremely sensitive to 0.5 mM manganese compared to the single mutants. (C) The microarray heat-map representing up- and downregulated genes. (D) Intracellular levels of glutamate and glutamine in the presence or absence of manganese stress. **, *P* < 0.01; ****, *P* < 0.0001. (E) A GFP reporter system (GFPmut3*) was used to determine the intracellular pH. The intracellular pH in the presence or absence of manganese, glutamine, or pGlnA plasmid were estimated. ****, *P* < 0.0001. The graphs were plotted from mean ± SD from six different experiments.

Glutamine synthetase (GlnA) utilizes Mn^2+^ as a cofactor (18). Our earlier finding has shown that the level of GlnA is increased in the manganese-fed E. coli Δ*mntP* strain (9). We observed that 8 mM and 1 mM manganese caused similar growth inhibition in WT and Δ*mntP* strains, respectively (see Fig. S2A in the supplemental material). This observation suggests that the degree of manganese toxicity in the 8 mM manganese-treated WT strain is similar to that of the 1 mM manganese-treated Δ*mntP* cells. Next, we performed Western blotting to show that the elevated GlnA levels were comparable in the WT and Δ*mntP* strains treated with 8 mM and 1 mM manganese, respectively (Fig. 1A). Further growth analysis reveals that 0.5 mM manganese was severely toxic to the Δ*mntP* Δ*glnA* strain in comparison to the Δ*mntP* or Δ*glnA* single mutants (Fig. 1B; see also Fig. S1 in the supplemental material). This toxicity is rescued by the presence of the plasmid pGlnA expressing functional *glnA* (Fig. S2B). Furthermore, we observed that the viability of the Δ*mntP* Δ*glnA* double mutant was 3-times less than that of the Δ*mntP* strain under a higher dose of manganese (10 mM), which was alleviated by expression of *glnA* from the pGlnA plasmid (Fig. S2C). These observations suggest that GlnA function is crucial for the Δ*mntP* strain under manganese shock.

To understand how *glnA* contributes to overcoming manganese toxicity, we performed microarray experiments using WT, Δ*glnA*, and Δ*mntP* Δ*glnA* mutants grown in the presence or absence of 1 mM manganese. The altered gene expressions with respect to the WT strain were identified and compared with our previously reported altered gene expression profile of the Δ*mntP* strain cells treated with 1 mM manganese (Fig. 1C) (9). While most of the genes for glutamate/aspartate or glutamine transporters (*gltIJKL*, *gltP*, and *glnHPQ*) were upregulated, the essential genes involved in glutamate-dependent acid resistance (e.g., *gadABC*) and nitrate/nitrite reductase pathways (*nap*, *nar*, *nir*, and *nrf* genes) were repressed in the manganese-fed Δ*mntP* strain (Fig. 1C). A modest level of repression of the genes in the nitrate/nitrite reductase pathways in the manganese-fed WT strain was also observed, suggestive of a mild effect of 1 mM manganese in the WT cells (Fig. 1C). These observations indicate that the intracellular pH homeostasis and nitrate/nitrite metabolism could be altered under manganese toxicity.

Conversely, expression of acid resistance, nitrate/nitrite metabolism, and ammonia transport (*amtB*) genes was upregulated in the Δ*glnA* strain (Fig. 1C). The genes that confer acid resistance, glutamate-glutamine cycling, and ammonia transport were further strongly activated in the Δ*mntP* Δ*glnA* strain and manganese-fed Δ*glnA* and Δ*mntP* Δ*glnA* strains (Fig. 1C). Interestingly, the expression of the genes in the nitrate/nitrite reductase pathways were sequentially decreased in the following order: Δ*glnA* strain > manganese-fed Δ*glnA* strain > Δ*mntP* Δ*glnA* strain > manganese-fed Δ*mntP* Δ*glnA* strain (Fig. 1C). These observations indicate that GlnA possibly alters nitrogen metabolism and pH homeostasis to alleviate manganese shock.

### GlnA-mediated glutamine synthesis is crucial for the increased cellular pH under manganese toxicity.

The glutamate-glutamine cycle generates glutamate and glutamine, which produce most of the nitrogenous metabolites (19). Glutamate and glutamine play pivotal roles in acid-resistant systems (20–22). We noted that the cellular pool of these two amino acids increased up to 3-fold in the manganese-treated Δ*mntP* strain (Fig. 1D). Since manganese stress blocks heme biogenesis (8), where glutamate acts as a precursor, we can argue that fluxes of glutamate to heme will be inhibited, causing an increased glutamate level under manganese stress. From this observation, we speculate that the activated GlnA (Fig. 1A and B) increased the cellular glutamine pool in the manganese-fed Δ*mntP* strain.

We have previously noticed that the cellular pH in the manganese-fed Δ*mntP* strain is elevated (9). In this study, we accurately determined intracellular pH in the unfed and manganese-fed strains of E. coli using a pH-sensitive GFPmut3*-bearing plasmid (23, 24). The Δ*mntP* mutant exhibited a cellular pH of 7.3 ± 0.18, and it was dramatically elevated to about pH 8.3 ± 0.09 under 1 mM manganese treatment (Fig. 1E). However, the cellular pH declined to 5.7 ± 0.27 and 5.5 ± 0.15 in the Δ*glnA* and Δ*mntP* Δ*glnA* strains, respectively (Fig. 1E). Further exposure to 1 mM manganese failed to elevate the cellular pH in the Δ*glnA* strain (Fig. 1E). The intracellular pH of the Δ*mntP* Δ*glnA* strain modestly increased to 6.1 ± 0.2 upon 1 mM manganese exposure (Fig. 1E). Intracellular pH of the WT strain was elevated from 7.4 ± 0.08 to 7.8 ± 0.05 and 8.4 ± 0.12 under 1 mM and 8 mM manganese exposure, respectively (Fig. 1E).

The Δ*glnA* strain was complemented with a plasmid bearing *glnA* (pGlnA) to show that the cellular pH was elevated to 7.4 ± 0.11, which was further raised to 7.9 ± 0.04 upon exposure to 1 mM manganese, a value similar to the pH of the 1 mM manganese-fed WT strain (Fig. 1E). Interestingly, expression of *glnA* from pGlnA alone could elevate pH to 8.4 ± 0.09 in the Δ*mntP* strain without any manganese shock (Fig. 1E). We also supplemented 4 mM glutamine to show that it elevated the intracellular pH to 8.1 ± 0.06 in the Δ*glnA* strain (Fig. 1E). These data additionally support the observation that the GlnA-mediated glutamine production plays a crucial role in elevating intracellular pH under manganese stress.

### Glutaminases catalyze ammonia production to raise cellular pH under manganese stress.

**FIG 2:**
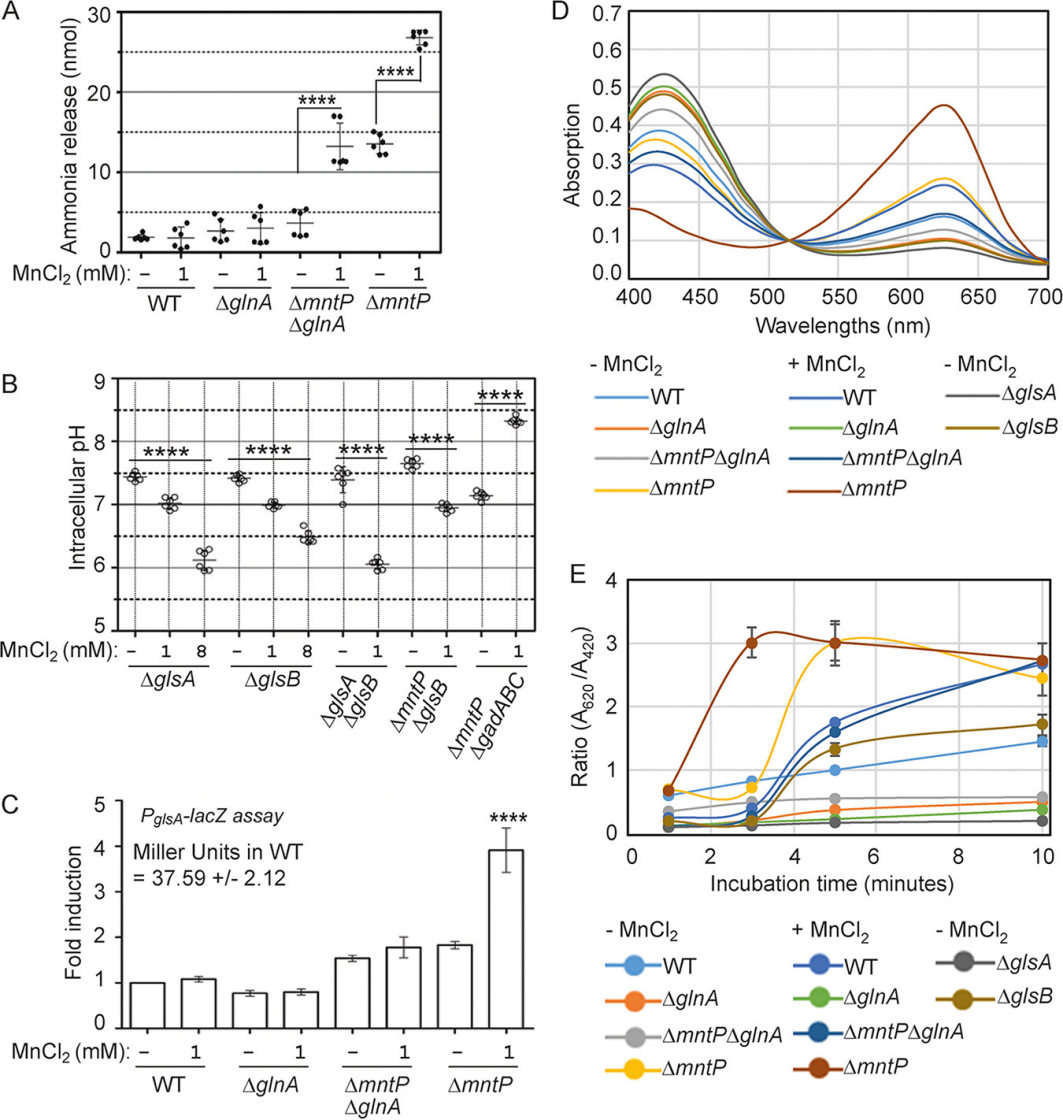
Ammonia liberation by GlsA/B raises intracellular pH. (A) Cellular ammonia release from the unfed and manganese-fed E. coli strains was estimated using ab83360 ammonia assay kit (Abcam) and plotted. ****, *P* < 0.0001. (B) Intracellular pH was measured and plotted for the E. coli strains grown in the presence or absence of manganese using GFPmut3* reporter. ****, *P* < 0.0001. (C) Relative activity of *glsA* promoter in the different E. coli strains grown in the presence or absence of manganese was plotted (using a *lacZ* reporter). The promoter activity in the WT strain was 37.59 ± 2.12 Miller units, which was normalized to 1 for fold change calculation. ****, *P* < 0.0001 against untreated WT control. (D) Absorption spectrums of bromocresol green pH indicator dye was recorded after allowing the cells to liberate ammonia. Note that the absorption at 620 nm sharply increased for the Δ*mntP* strain treated with manganese at the 3-min time point (also see Fig. S5A in the supplemental material for all time points). (E) *A*_620_/*A*_420_ of bromocresol green dye was obtained and plotted to show the kinetics of ammonia release from different strains grown in the presence or absence of manganese.

From the involvement of GlnA and the upregulation of *amtB* in the Δ*glnA* and Δ*mntP* Δ*glnA* strains (Fig. 1C), we speculate that a likely elevation of ammonia levels might be a factor contributing to the surge in pH under manganese stress. Indeed, the cellular ammonia level was found to be increased in the Δ*mntP* strain (Fig. 2A). Manganese supplementation further elevated ammonia in the Δ*mntP* strain (Fig. 2A). The ammonia levels in the Δ*glnA* and Δ*mntP* Δ*glnA* strains remained at basal WT level (Fig. 2A). Interestingly, 1 mM manganese treatment raised the ammonia level in the Δ*mntP* Δ*glnA* strain (Fig. 2A), plausibly due to the sole utilization of cellular glutamine through the GlsA/B enzymes to produce glutamate and ammonia, as manganese treatment inactivates GOGAT to produce glutamate (9). The last observation also explains the modestly elevated pH in the manganese-fed Δ*mntP* Δ*glnA* strain (Fig. 2A).

The GlsA and GlsB enzymes break down glutamine to produce glutamate, releasing ammonia (21). We assessed that Δ*glnA* Δ*glsA*, Δ*mntP* Δ*glsA*, and Δ*mntP* Δ*glnA* Δ*glsA* strains grow poorly compared to Δ*glnA*, Δ*glsA*, and Δ*mntP* single mutant strains on LB agar (see Fig. S3 in the supplemental material). Besides, the double and triple mutants formed partially lysed cell pellets while growing in LB broth. The Δ*glnA* Δ*glsB* and Δ*mntP* Δ*glnA* Δ*glsB* mutants also grew poorly on LB agar (Fig. S3), but they were not lysed in LB broth. The Δ*mntP* Δ*glsB* strain grew optimally both on LB agar and LB broth. These observations indicate that the GlsA function is more critical than GlsB in the Δ*mntP* mutant.

We found that the Δ*glsA*, Δ*glsB*, and Δ*mntP* Δ*glsB* strains exhibited an optimum physiological pH. 1 mM manganese modestly declined cytoplasmic pH to 7.0 ± 0.09 (Fig. 2B). In the Δ*glsA* and Δ*glsB* mutants, 8 mM manganese lowered the pH to 6.1 ± 0.16 and 6.5 ± 0.1 respectively, (Fig. 2B). Conversely, 1 mM manganese was enough to drop the intracellular pH to 6.1 ± 0.08 in the Δ*mntP* Δ*glsB* strain. Why manganese stress caused the pH drops in Δ*glsA*, Δ*glsB*, and Δ*mntP* Δ*glsB* mutants is discussed later. These observations indicate that manganese fails to elevate the intracellular pH in the absence of GlsA or GlsB. The glutamate-dependent acid-resistant (*gad*) system did not play any role in alkalization in the Δ*mntP* mutant as is evident from the observation that the Δ*mntP* Δ*gadABC* mutant successfully elevated the pH to 8.3 ± 0.06 (Fig. 2B).

To check whether *glsA* was upregulated under manganese stress, we constructed a *glsA-lacZ* transcriptional fusion (P*_glsA_*-*lacZ*) and integrated it into the genome of the strains under study. The Δ*mntP* and Δ*mntP* Δ*glnA* strains exhibited an about 1.7-fold increase in β-galactosidase activity compared to that of the WT counterpart (Fig. 2C). Upon exposure to 1 mM manganese, the Δ*mntP* strain, but not the Δ*mntP* Δ*glnA* strain, exhibited a 3.7-fold increase in β-galactosidase activity (Fig. 2C). The *glsA-lacZ* activity remained unaltered in unfed and manganese-fed Δ*glnA* strain cells (Fig. 2C).

We performed an ammonia production assay as a function of GlsA activity using the bromocresol green pH indicator dye (25). Instead of merely visualizing color changes (see Fig. S4A in the supplemental material) as described (25), we recorded the absorption spectrum of the dye (Fig. S4B). A gradual increase in the ratio of the two absorption peaks (*A*_620_/*A*_420_) of the dye highly correlated with the increasing amounts of an added base to raise the pH from 3.2 to 5.9 (Fig. S4C). Thus, we consider the GlsA-catalyzed ammonia base liberation from E. coli strains as a function of *A*_620_/*A*_420_.

A steep increase in *A*_620_ and *A*_620_/*A*_420_ values was observed within 3 min when the indicator dye was incubated with unfed Δ*mntP* or manganese-fed Δ*mntP* strain cells (Fig. 2D and E), indicating a faster ammonia release under this condition. As expected, the *A*_620_/*A*_420_ ratio suggested that the Δ*glsA* strain did not liberate any ammonia with time (Fig. 2E). Similarly, from the *A*_620_/*A*_420_ ratio, it appeared that unfed and manganese-fed Δ*glnA* and unfed Δ*mntP* Δ*glnA* strains also did not liberate ammonia, indicating that GlsA function in these strains was compromised (Fig. 2D and E). However, the manganese-fed Δ*mntP* Δ*glnA* mutant raised the *A*_620_/*A*_420_ value to some extent, indicating a moderate GlsA activity in this condition (Fig. 2E). This observation is consistent with the finding that intracellular ammonia levels increased in the manganese-fed Δ*mntP* Δ*glnA* strain (Fig. 2A). Unfed WT and Δ*glsB* strains increased the *A*_620_/*A*_420_ ratios very slowly, while the manganese-fed WT strain moderately elevated the *A*_620_/*A*_420_ values throughout the incubation (Fig. 2E). We attempted to correlate ammonia liberation as a function of pH changes. However, unlike alterations in *A*_620_/*A*_420_ with the increasing amounts of added bases, the alterations in pH units were negligible (see Fig. S5 in the supplemental material), as bromocresol green dye has a pK_a_ value of ~5.0 (Fig. S4).

### Intracellular alkalization dictates manganese-driven riboswitch activation.

**FIG 3:**
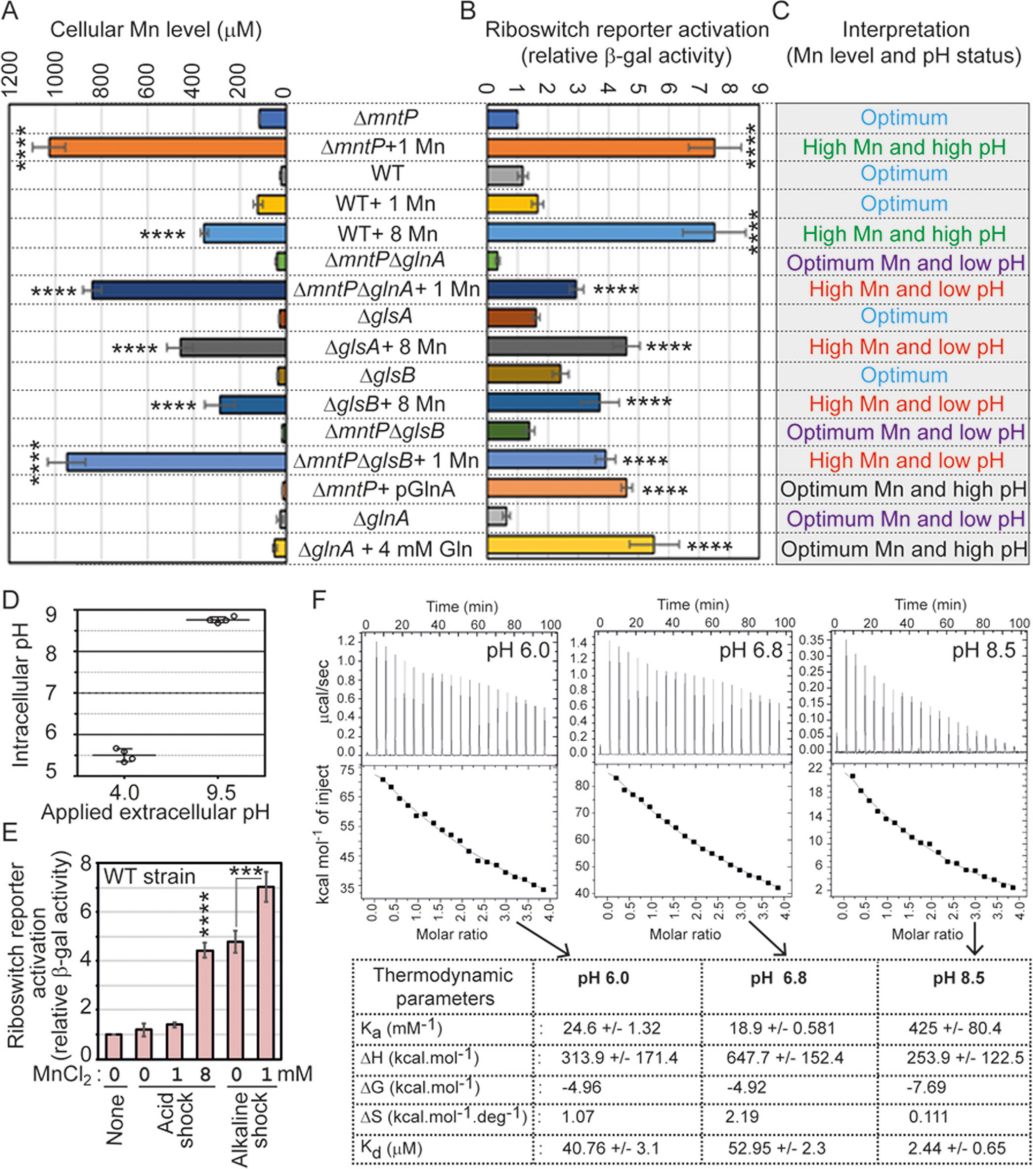
The concerted action of cellular manganese and alkaline pH to activate the *mntP* riboswitch. (A) Bar diagram represents the estimated levels of intracellular manganese in the untreated and treated E. coli strains (using ICP MS methodology). ****, *P* < 0.0001 against the untreated Δ*mntP* strain. (B) The riboswitch reporter activities in the different strains grown under specific conditions were plotted from the *lacZ* reporter (relative β-gal activity) assays. The riboswitch reporter activity in the Δ*mntP* strain was 16.62 ± 1.62 Miller units, which was normalized to 1 for the fold change calculation. ****, *P* < 0.0001 against the untreated Δ*mntP* strain. (C) Combining the cellular pH (Fig. 1E and 2B) and manganese levels (Fig. 3A) in a specific strain grown under desired conditions, we classified intracellular environments into five different categories. (D) Intracellular pH of WT E. coli strain was determined under the specific acid and alkaline conditions. (E) Relative riboswitch reporter activity was estimated in the WT E. coli strain under acid and alkaline condition and in the presence or absence of manganese. The riboswitch reporter activity in WT strain was 16.84 ± 1.01 Miller units, which was normalized to 1 for the fold change calculation. ***, *P* < 0.001; ****, *P* < 0.0001 against untreated WT strain. (F) ITC experiments were performed under 3 different pH, *viz.*, 6.0, 6.8, and 8.5 to get binding parameters for the *mntP* riboswitch element and manganese interaction.

We determined the cellular manganese concentrations in E. coli strains (Fig. 3A). The 1 mM manganese-fed Δ*mntP* strain retained the highest level of manganese (Fig. 3A). WT cells treated with 1 mM exogenous manganese elevated the cellular level of manganese to the extent that was equivalent to the manganese content in the unfed Δ*mntP* strain (Fig. 3A). Exogenous 8 mM manganese raised the cellular manganese level from 22 μM to 400 μM in the WT cells (Fig. 3A). Since 1 mM and 8 mM manganese conferred similar phenotypes to the Δ*mntP* and WT strains, respectively (Fig. 1A and E; Fig. S2A), we suppose that ~400 μM would be a threshold level of cellular manganese that confers the maximum degree of toxicity in E. coli. We estimated that the 1 mM manganese-fed Δ*mntP* Δ*glnA* and Δ*mntP* Δ*glsB* strains and the 8 mM manganese-fed Δ*glsA* and Δ*glsB* strains accumulated more than or equal to the threshold level of manganese (Fig. 3A).

Recently, a *lacZ* reporter construct has been used to demonstrate the riboswitch activity in an *mntP*-deficient PM1205 E. coli strain under manganese shock (11). Similarly, we constructed a P_T7A1_-5′-UTR*_mntP_*-*lacZ* reporter. Consistent with the observation (11), our reporter construct indicated a 7.5-fold increase in β-galactosidase activity in 1 mM manganese-fed and 8 mM manganese-fed Δ*mntP* and WT strains, respectively (Fig. 3B).

Combining the levels of cellular manganese (Fig. 3A) and pH (Fig. 1E and Fig. 2B) in E. coli strains, we categorized intracellular environments as follows: (i) optimum, when intracellular manganese was below the threshold level and pH remained around 7.4; (ii) high Mn and high pH, when intracellular manganese was equal to or above the threshold level and pH elevation was above 8.0; (iii) high Mn and low pH, when intracellular manganese was equal to or above threshold level but pH declined to be acidic; (iv) optimum Mn and high pH, when intracellular manganese remained at optimum but pH elevation was above 8.0; and (v) optimum Mn and low pH, when intracellular manganese remained at optimum but pH was declined to be acidic (Fig. 3C). As expected, the “high Mn and high pH” category (1 mM and 8 mM manganese-fed Δ*mntP* and WT strains, respectively) showed the highest level of riboswitch reporter activation (Fig. 3B and C). Interestingly, in the “high Mn and low pH” category (represented by 1 mM manganese-fed Δ*mntP* Δ*glnA* and Δ*mntP* Δ*glsB* strains or 8 mM manganese-fed Δ*glsA* and Δ*glsB* strains), the riboswitch reporter activity remained partial compared to the “high Mn and high pH” category (Fig. 3B and C). Similarly, in the “optimum Mn and high pH” category (represented by the Δ*mntP* strain harboring pGlnA and Δ*glnA* strain fed with 4 mM glutamine), the riboswitch reporter was activated substantially but not to the highest level (Fig. 3B and C). This data suggests that both alkalization or manganese toxicity can partially activate the riboswitch reporter, but to achieve the highest level of activation, concerted action of manganese and cytoplasmic alkalization was required. To recreate this scenario, we grew the WT strain under acidic or alkaline shock (pH 4.0 or 9.5; unbuffered) so that the cellular pH equilibrated to 5.5 or 8.6, respectively (Fig. 3D), and subsequently observed the effect of supplemental manganese on the riboswitch reporter activation. Bolstering our finding, the alkaline pH, or the toxic level of manganese at acidic pH could partially activate the riboswitch reporter, but the combination of both activated the riboswitch reporter to the maximal level (Fig. 3E). We also performed the riboswitch reporter activity in different pH (buffered) media in the presence or absence of manganese in the Δ*mntP* strain. Consistently, high pH (8.5) alone could activate the riboswitch reporter to 5-fold, which further increases to 9-fold in the presence of manganese (see Fig. S6A in the supplemental material). However, low pH (pH 5.0) and neutral pH (pH 7.0) media could not activate the riboswitch reporter sufficiently in the presence of 1 mM manganese (Fig. S6A). These data directly establish that both alkaline pH and manganese have additive effects in the activation of the *mntP* riboswitch. As a negative control, we used the P_T7A1_-*lacZ* reporter to show that the promoter activity was not influenced by pH variations (Fig. S6B).

Similarly, we incorporated the *alx* riboswitch reporter (P_T7A1_-5′-UTR*_alx_*-*lacZ* reporter) in the Δ*mntP* strain to show that 1 mM and 2 mM exogenous manganese activated the *alx* riboswitch reporter to 3- and 6.5-folds, respectively, in unbuffered LB broth, where manganese treatment could intrinsically raise the pH (see Fig. S7 in the supplemental material). This manganese-mediated riboswitch reporter activation was dramatically suppressed in buffered LB broth at pH 5.0 (Fig. S7). However, the riboswitch reporter activation was modest (3.5-fold under 2 mM manganese stress) in buffered LB broth at pH 7.0 (Fig. S7). Remarkably, the *alx* riboswitch reporter was activated at the highest level in the presence of manganese in buffered LB broth at pH 8.5 (Fig. S7). Interestingly, pH 8.5 alone could activate the riboswitch reporter to 3.5-folds (Fig. S7). These data support the previous reports that either alkaline pH or manganese can activate the *alx* riboswitch reporter (11, 16, 17). Herein, we show that the cumulative effect of both alkaline pH and manganese is required for *mntP* and *alx* riboswitch reporter activation at the highest degrees.

We conducted isothermal titration calorimetry (ITC) experiments to address whether the alkaline pH sensitizes the interaction between manganese and the riboswitch element. For this study, we generated the whole 5′-UTR of *mntP* using T7 RNA polymerase-based *in vitro* transcription. The RNA was dissolved in buffers with pH 6.0, 6.8, and 8.5 to equilibrate, and 4 μM RNA was titrated by increasing concentrations of MnCl_2_ at 25°C. The ITC profiles exhibited an entropy-driven reaction at pH 6.0, 6.8, and 8.5 (Fig. 3F). Interestingly, a lower Gibbs free energy (Δ*G*) was recorded for the binding assay at pH 8.5 (−7.69 kcal/mol) than at pH 6.0 and 6.8 (−4.96 and −4.92 kcal/mol, respectively). Conversely, the dissociation constant (*K_d_*) value was lowest at pH 8.5 (2.44 ± 0.65 μM) compared to *K_d_* values at pH 6 and pH 6.8 (40.76 ± 3.1 and 52.95 ± 2.3 μM, respectively). Thus, a substantially lower Gibbs free energy (Δ*G*) and 17- to 22-fold lower *K_d_* values at alkaline pH suggested that manganese binds to the riboswitch element more favorably and tightly under alkaline condition.

## DISCUSSION

The current study unfolds three hitherto unknown aspects of bacterial physiology. First, we show that manganese intoxication triggers alkalization of cytoplasm, mainly by altering nitrogen metabolism (Fig. 1). Second, by decoupling the intracellular enlarged manganese pool from intrinsic alkalization, we demonstrate that the concerted actions of these two factors activate the *mntP*, and *alx* riboswitch reporters maximally (Fig. 2 and 3; see also Fig. S7 in the supplemental material). Third, we reveal how intelligently E. coli calibrates its metabolism under manganese stress, thereby causing intrinsic cellular alkalization, which is critical for the riboswitch-mediated manganese homeostasis (Fig. 1 and Fig. 3A). Interestingly, alkaline pH inhibits MntH-mediated manganese import in Salmonella enterica (26). Since E. coli and S. enterica MntH proteins are 94% identical, it is quite plausible that the manganese-induced alkalinity in E. coli would also inhibit MntH function. Interestingly, the foundational study on the *mntP* riboswitch has been performed using a mutant strain of E. coli (that makes MntP nonfunctional) (11). Using this mutant strain, they found that 1 mM manganese is enough to cause riboswitch reporter activation (Fig. 3B). Consistent with this, our study with the Δ*mntP* strain also shows riboswitch reporter activation at 1 mM manganese. However, in addition, we also used a WT strain of E. coli to show that 8 mM manganese is required to maximally activate the riboswitch reporter (Fig. 3B).

**FIG 4:**
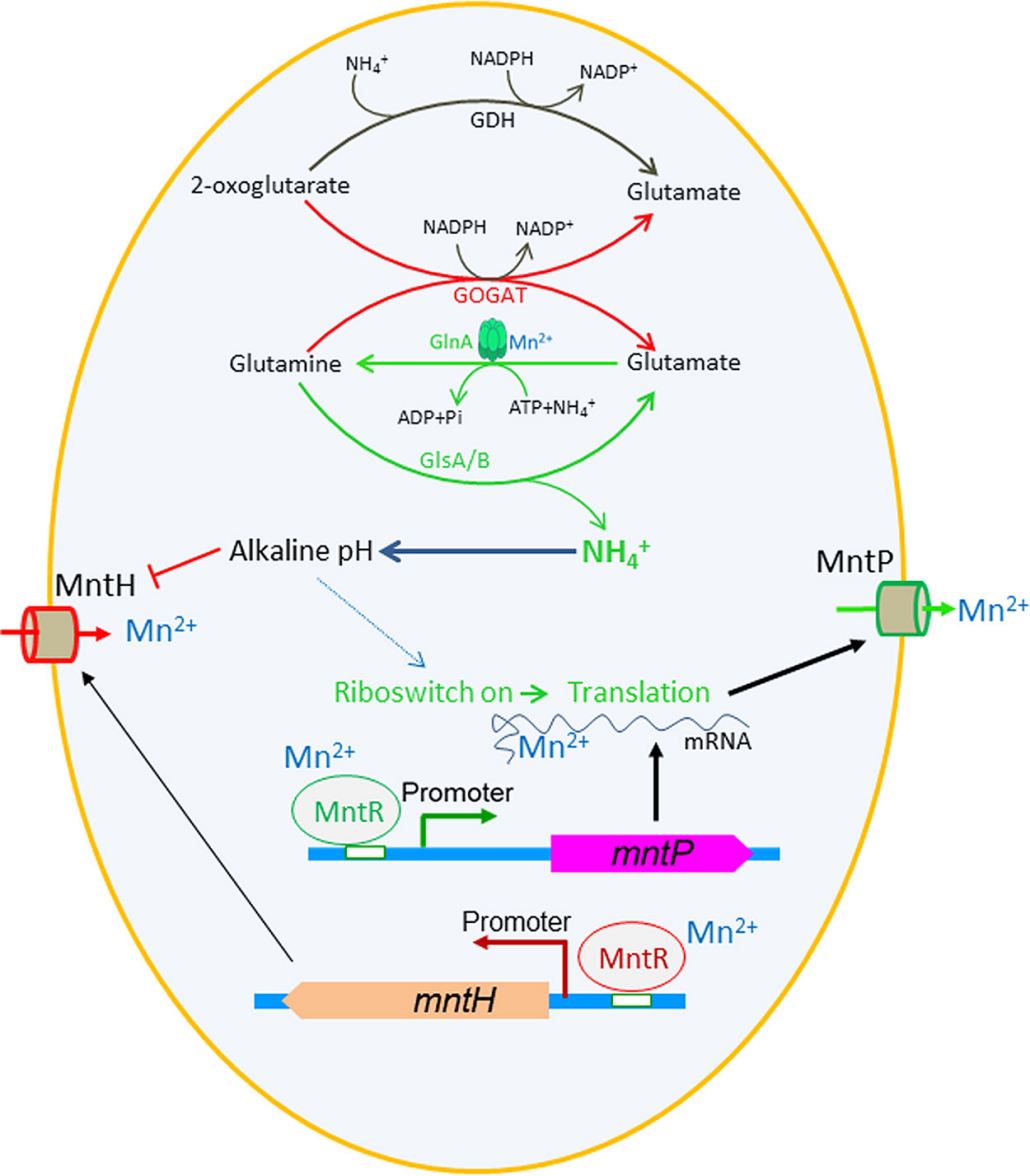
The integrated model depicts how the intrinsic cellular alkalization contributes to manganese homeostasis. Skewed functioning of the glutamate-glutamine cycle enzymes (*viz.*, activation of GlnA and GlsA/B enzymes and deactivation of GOGAT) under manganese stress leads to ammonia production and subsequent alkalization. The alkaline pH favors interaction between manganese and the riboswitch element to upregulate MntP expression enhancing manganese efflux. The excess manganese activates MntR to repress MntH importer. The alkaline pH also blocks MntH functioning, as reported (26), to resist manganese intoxication.

The glutamate-glutamine cycle plays a pivotal role in acid resistance (20–22). While GlnA solely synthesizes glutamine using glutamate, two alternative pathways exist for glutamine to glutamate production. The principle one is catalyzed by GOGAT (27, 28). The other one is GlsA/B-mediated glutamine degradation, generating glutamate and ammonia (21, 29). We have previously demonstrated that manganese toxicity inactivates GOGAT (9). Thus, glutamate production via activated GlsA/B under manganese shock (Fig. 2C to E) liberates ammonia, raising the cellular pH (Fig. 4). Conversely, a complete shutdown of glutamine to glutamate production in manganese-fed Δ*glsA*, Δ*glsB*, and Δ*mntP* Δ*glsB* mutants might affect glutamate-mediated acid resistance systems that help to raise the pH, thereby lowering cellular pH (Fig. 2B). We also tested the role of glutamate dehydrogenase (*gdhA*) that catalyzes glutamate synthesis, conjugating α-ketoglutarate, and ammonia in pH elevation. However, deleting *gdhA* from the Δ*mntP* strain barely alters the growth profile, or cellular pH, under manganese stress (see Fig. S8 in the supplemental material), suggesting that *gdhA* plays no role under manganese stress.

Previously, GlsA, a glutaminase that functions under acidic pH, and glutamine have been shown to constitute an acid resistance system that accelerates ammonia production to cope with external acid shock (21, 29). However, whether ammonia production induced by GlsA action could intrinsically cause intracellular alkalization was not envisioned. Furthermore, no relevant physiological importance of GlsB, which functions optimally at alkaline condition, is known so far (21, 29). The present study reveals that GlnA and GlsA/B work in concert, causing intrinsic alkalization (i.e., without extrinsic acid-shock stimulus) in E. coli under manganese stress. Thus, for the first time, our study provides a rationale for the existence of two Gls proteins in E. coli (21, 29). A recent report suggests that deletion of glutamine synthetase (GS) in Leishmania donovani affects its viability (30). Mycobacterium tuberculosis GS has also been considered as a potential therapeutic target to inhibit its growth (31). Whether glutamate-glutamine cycle-mediated alkalization imparts viability of these pathogens needs further investigation.

In humans, excess manganese dents the metabolic processes in basal ganglia causing manganism (32, 33). Besides, glutamine synthetase (GS) plays a pivotal role in recycling the neurotransmitter glutamate and in ammonia detoxification, thereby preventing glutamate/ammonia neurotoxicity. Alteration in the expression of GS contributes to pathologies of several neurological disorders, including hepatic encephalopathy, ischemia, amyotrophic lateral sclerosis, epilepsy, Alzheimer’s and Parkinson’s diseases, traumatic brain injury, etc. (34). Glutaminases (Gls) have been reported to be involved in neuroinflammation responses of various neurological disorders (35). Thus, in neurodegenerative diseases, glutamine, ammonia, and glutamate play significant roles (36). Therefore, considering the universality of the key biochemical reactions, the elevation of pH may play critical roles in manganism and other neurological disorders.

The alkaline pH favored the interaction between manganese and the *mntP* riboswitch element by decreasing the Δ*G* and increasing *K_a_* (Fig. 3F). Corresponding dissociation constant (*K_d_*) values for this interaction were 40.76 ± 3.1 μM, 52.95 ± 2.3 μM, and 2.44 ± 0.65 μM at pH 6.0, 6.8, and 8.5, respectively (Fig. 3F). These values are well within the range of basal levels of manganese (20 to 30 μM) in E. coli (9). Specifically, the *K_d_* value 2.4 μM at pH 8.5 is much lower than the basal level of manganese. This observation explains how alkaline pH sensitized the riboswitch activation even in the presence of basal levels of manganese (Fig. 3A). Since alkaline pH has the ability to break up some Hoogsteen base pairs (37), a similar scenario for *mntP* riboswitch could facilitate further manganese binding. Especially the extended 5′-UTR portion downstream of the manganese-binding aptamer (11) could fold back to form a triple helix (38), inhibiting manganese binding at a lower pH. A smaller Δ*S* value at alkaline pH possibly indicates that manganese is required to make lesser disorder at alkaline pH than at acidic pH to turn the riboswitch on (Fig. 3F).

The *K_d_* value at pH 6.8 for the *mntP* riboswitch in our study (52.95 ± 2.3 μM) matches well with the reported *K_d_* values for other manganese-sensing *yybP*-*ykoY* riboswitches from Lactococcus lactis and Streptococcus pneumoniae (30 to 40 μM and 54 ± 26 μM, respectively) (39, 40). In contrast to our finding, a previous report has shown a *K_d_* value of 7 nM for manganese-sensing *mntP* riboswitch aptamer from E. coli at pH 6.8 (41). This low nanomolar *K_d_* value is far below the intracellular concentration of manganese in WT E. coli (20 to 30 μM) (9). Therefore, this value appears to be physiologically irrelevant, as the *mntP* riboswitch would have always been activated at that cellular level of manganese. In other words, the riboswitch activation would not have required a further increase in cellular manganese level. Furthermore, where the authors have shown an enthalpy-driven reaction (41), we observed an entropy-driven reaction for the manganese and *mntP* riboswitch interaction (Fig. 3F). The length of the riboswitch sequences in our study (whole 5′-UTR) and in the previous report (shorter riboswitch aptamer) (41) might be the possible reason for such observed discrepancies.

## MATERIALS AND METHODS

### Bacterial strains, plasmids, media, and growth conditions.

The wild-type BW25113 (WT) and the KEIO knockout mutant strains of E. coli used in this study are listed (Table 1). The knockouts were verified by PCR, freshly transduced into the WT background by P1 phage, and sequenced to confirm the deletion. The double and triple mutants were generated by P1 phage transduction. The P*_glsA_*-*lacZ* transcriptional fusion construct was made by fusing the entire *glsA* promoter region beginning with 220 nucleotides upstream of the transcription site and the 68-nucleotide 5′-UTR of *glsA* to *lacZ*. Similarly, we constructed two more translational fusion reporters, P_T7A1_-5′-UTR*_mntP-_*_17-codons_-*lacZ* and P_T7A1_-5′-UTR*_alx-_*_17-codons_-*lacZ*. For this, the entire 5′-UTR and the first 17 codons of the *mntP* or *alx* genes were fused with *lacZ*. Further fusion PCRs were performed to attach the T7A1 promoter upstream of these reporters. We also made the P_T7A1_-*lacZ* reporter a negative control to check the effects of different pH on T7A1 activity. The reporter constructs were cloned into the pAH125 vector (42). The cloned constructs were then integrated into the genome of WT strain using the pINT helper plasmid and screened for single integrant, as described (42). The genotype was transduced to various desired strains of E. coli by P1 transduction. The gifted and constructed plasmids in this study are mentioned in Table 1. The oligonucleotides used in the study are mentioned in Table S1 in the supplemental material. All studies were done in LB broth and LB agar media at 37°C.

**TABLE 1:** List of strains and plasmids used in this work

Strain or plasmid	Description/feature(s)^*a*^	Reference or source
Strain		
BW25113	Escherichia coli; *rrnB3*Δ*lacZ4787hsdR514*Δ(*araBAD*) 567 Δ(*rhaBAD*)568 *rph-1*	46
Δ*mntP* mutant	BW25113, Δ*mntP*::Kan^r^	46
Δ*glnA* mutant	BW25113, Δ*glnA*::Kan^r^	46
Δ*glsA* mutant	BW25113, Δ*glsA*::Kan^r^	46
Δ*glsB* mutant	BW25113, Δ*glsB*::Kan^r^	46
Δ*gadA* mutant	BW25113, Δ*gadA*::Kan^r^	46
Δ*gadB* mutant	BW25113, Δ*gadB*::Kan^r^	46
Δ*gadC* mutant	BW25113, Δ*gadC*::Kan^r^	46
Δ*glsA* Δ*glsB* mutant	BW25113, Δ*glsA*, Δ*glsB*::Kan^r^	This study
Δ*mntP* Δ*glnA* mutant	BW25113, Δ*mntP*, Δ*glnA*::Kan^r^	This study
Δ*mntP* Δ*glsA* mutant	BW25113, Δ*mntP*, Δ*glsA*::Kan^r^	This study
Δ*mntP* Δ*glsB* mutant	BW25113, Δ*mntP*, Δ*glsB*::Kan^r^	This study
Δ*mntP* Δ*glnA* Δ*glsA* mutant	BW25113, Δ*mntP*, Δ*glnA*, Δ*glsA*::Kan^r^	This study
Δ*mntP* Δ*glnA* Δ*glsB* mutant	BW25113, Δ*mntP*, Δ*glnA*, Δ*glsB*::Kan^r^	This study
Δ*mntP* Δ*gadABC* mutant	BW25113, Δ*mntP*, Δ*gadA*, Δ*gadB*, Δ*gadC*::Kan^r^	This study
Plasmid		
pET28a (+)	Kan^r^; T7-promoter; IPTG inducible	Novagen
pET-glnA	*glnA* cloned in NdeI and HindIII sites of pET28a (+) vector	This study
pCL1920	Amp^r^; Lac-promoter, IPTG inducible	A gift from J. Gowrishankar (47)
pAH125	Kan^r^; LacZ reporter	A gift from R. Chaba (42)
pINT	Amp^r^; λ-integrase	A gift from R. Chaba (42)
pBAD-GFPmut3*	Amp^r^; Lac-promoter, arabinose inducible	C. Mullineaux (23)
pGlnA	pCL1920 with *glnA* at NdeI and HindIII	This study
pBAD/*Myc*-His A	Amp^r^; cloning vector	Invitrogen
pDAK1	pBAD/*Myc*-His A; two NdeI sites were mutated and NcoI site was replaced by NdeI	This lab
pGlsA	pDDAK1 with *glsA* at NdeI and HindIII	This study
pGlsB	pDDAK1 with *glsB* at NdeI and HindIII	This study

a Kan^r^, kanamycin resistance; Amp^r^, ampicillin resistance; Cm^r^, chloramphenicol resistance.

### Growth curve analysis.

The primary culture was grown overnight at 37°C with shaking. For growth analysis, the cultures were diluted to 0.6 optical density at 600 nm (OD_600_), and 1% of the diluted culture was inoculated in 1 mL of LB broth in the presence and absence of desired concentrations of manganese. Growth curve analysis was done using a Bioscreen C growth analyzer (Oy growth curves Ab Ltd).

### Viability assay.

To check the survivability under extreme manganese stress, 1% overnight cultures were inoculated in LB media supplemented with 10 mM manganese. After incubating for the indicated times, the cultures were diluted as required and plated on the LB agar surface. The number of colonies were counted and compared with the initial number of cells determined from the untreated counterparts.

### Microarray experiments.

The saturated overnight cultures of E. coli strains were inoculated in the fresh LB medium at 1:100 dilution and grown initially for 1 to 1.5 h to get the OD to about 0.3 and then grown again for 2.5 h in the presence or absence of 1 mM manganese at 37°C before harvesting the cell pellets. The pellets were washed with normal saline (0.9%) and stored by dissolving in RLT buffer (Sigma) before further processing. The microarray was done on an Agilent-based customized platform from Genotypic Technology, Bangalore. On average, three probes were designed for each gene. RNA extraction, quality control, microarray labeling, hybridization, scanning, and data analyses were done as mentioned previously (42).

### Estimation of intracellular glutamine and glutamate levels.

The WT and Δ*mntP* strain cells were grown in the presence or absence of 1 mM manganese. The cell pellets were washed with phosphate-buffered saline (PBS) and resuspended in 50% cold methanol. Subsequently, cells were subjected to three freeze-thaw cycles in liquid nitrogen followed by sonication. The supernatants were collected and dried using a SpeedVac vacuum concentrator. Liquid chromatography-tandem mass spectrometry (LC-MS/MS) analyses and quantification were done at the metabolomics facility, National Institute of Plant Genome Research, New Delhi, India, as per protocol (43). The amino acid levels were expressed as nanomoles per milligram of wet cell pellet weights.

### Estimation of intracellular pH.

The intracellular pH was estimated as described (24). Briefly, 1% of overnight primary cultures of the desired strains of E. coli harboring the pAra-TorA-GFPmut3* plasmid (23, 24) were inoculated in 200 mL of fresh LB broth to achieve an OD_600_ of 0.125. The cells were grown in the presence or absence of supplemented manganese or glutamine (at desired concentrations indicated in Results) for 2 h, and then green fluorescent protein (GFP) was induced with 0.003% L-arabinose. A total of 0.2 mM isopropyl-β-d-thiogalactopyranoside (IPTG) was used to induce GlnA from pGlnA-complemented strains. The cells were grown for another 2 h at 37°C after induction. The individual bacterial cell pellets were collected and washed with 1× PBS. The cell pellets were divided into 7 equal portions. Six portions of pellets were permeabilized by 20 mM sodium benzoate and incubated with 6 different buffers (pHs were 5.0, 6.0, 7.0, 7.5, 8.0, and 8.5) for 10 min so that the intracellular pH was equilibrated with applied external pH. GFP excitation was measured from 480 to 510 nm using an emission wavelength of 545 nm, and the standard curves were generated as described (24). The remaining cell pellet was dissolved in PBS to measure the GFP fluorescence directly. The intracellular pH was determined from the standard curve equations.

### β-galactosidase assay.

For the β-galactosidase assay, 1% of the overnight culture of bacterial strains were inoculated in fresh LB broth (initial OD_600_ was 0.125) and grown in the presence or absence of 1 mM manganese for 4 h at 37°C. The cell pellets were washed two times with Z-buffer (60 mM Na_2_HPO_4_, 40 mM NaH_2_PO_4_, 10 mM KCl, and 1 mM MgSO_4_), and the β-galactosidase assay was performed as described (44). Background β-galactosidase activity from the reporterless isogenic E. coli strains was determined and subtracted.

For the β-galactosidase assay, under exogenous alkaline and acid shocks were performed as described below. E. coli was grown in LB broth for 3 h at 37°C and then subjected to alkaline or acid shock. For this, the culture pH was adjusted to either 9.5 (using 4 M NaOH) or 4.0 (using 1 M HCl) and grown for 1 h at 37°C. In one set of assays, we measured the intracellular pH. In another set of assays, we performed β-galactosidase assays to know the riboswitch reporter activity. The riboswitch reporter activities were also checked by growing the E. coli cells in different buffered (pH 5.0, 7.0, and 8.5) LB broth.

### Western blotting experiments.

Untreated and manganese-treated cell pellets were harvested, washed with 1× PBS, and then lysed with B-PER bacterial protein extraction reagent (Thermo Scientific) followed by sonication. The total protein level was estimated by using the Bradford assay kit (Bio-Rad). Thirty micrograms of total cellular proteins from the individual samples were subjected to SDS-PAGE. The proteins were transferred to nitrocellulose membrane and stained with Ponceau S to visualize the resolution and equal loading in the PAGE. Western blotting was performed using polyclonal rabbit primary antibodies raised against purified GlnA protein and horseradish peroxidase (HRP)-conjugated secondary goat anti-rabbit polyclonal antibodies (Sigma-Aldrich). The blots were developed by Immobilon Forte Western HRP substrate (Millipore).

### RNA synthesis by *in vitro* transcription.

The template DNA for *in vitro* transcription was prepared by fusing the T7 promoter and the *mntP* riboswitch element including 357 bases. The RNA was synthesized using the T7 RiboMax express large-scale RNA production system (Promega). To remove the DNA template, the RQ1 RNase-free DNase was added to the reaction mixture to a concentration of 1 unit per microgram of template DNA and incubated for 15 min at 37°C. The reaction mixture was then extracted with phenol-chloroform and ethanol precipitated. The eluted RNA was then passed through the P-6 column to remove the unincorporated nucleotides.

### Isothermal titration calorimetry.

A MicroCal Auto-iTC200 calorimeter (MicroCal Inc.) was used for calorimetric measurements to probe the interaction of the *mntP* riboswitch element with manganese at different pH values. Prior to the ITC experiments, the *mntP* riboswitch RNA was equilibrated in the ITC buffers (30 mM HEPES [for pH 6 and 6.8] or 30 mM Tris-HCl [for pH 8.5], 150 mM NaCl) and then heated to 95°C for 2 min and placed on ice for 10 min. Then, 5 mM MgCl_2_ was added to the RNA solutions and incubated at 21°C for 15 min. The RNA samples were centrifuged for 20 min at 13,000 rpm. MnCl_2_ was dissolved in ITC buffers and filtered. Experiments were performed with 4 μM RNA (cell) and 75 μM MnCl_2_ (syringe) at 25°C. MnCl_2_ was also injected into the respective ITC buffers to get background enthalpy changes and subtracted from the experimental binding data. Data were fitted using a one-site binding model fixing stoichiometry (N) at 2 using MicroCal Origin software. Individual ITC experiments were performed 3 times.

### Ammonia liberation assay.

The ammonia liberation from E. coli strains was estimated using the ab83360 ammonia assay kit (Abcam). As mentioned previously, the cells were grown before harvesting the pellets. Pellets were washed with cold 1× PBS. Ten milligrams of cells was resuspended in 100 μL of assay buffer. The samples were then centrifuged at 13,000 rpm for 5 min at 4°C. The supernatant was collected in a clean tube and kept on ice. Fifty microliters of reaction mix was added to standards and the sample wells in a 96-well plate and incubated in the dark at 37°C for 60 min. The plate reading was immediately taken at OD_570_ in a microplate reader.

### The ammonia production assay (glutaminase A activity assay).

To assess the activity of the GlsA enzyme, different E. coli strains were grown in the presence and absence of manganese. The cells were then pelleted and washed with 1× PBS. The pellets were resuspended in 1× PBS by normalizing according to their respective weight. Three hundred microliters of cell suspension (approximately 30 mg of wet cell weight) was further taken from each sample and centrifuged. The pellets were dissolved in 200 μL of GlsA assay solution containing 1 g/liter l-glutamine, 0.25 g/liter bromocresol green, 90 g/liter NaCl, and 3 mL/liter Triton X-100. The pH was adjusted to 3.2 with HCl. The resuspended cell pellets were mixed thoroughly and incubated at 37°C for 1, 3, 5, and 10 min. We recorded the color changes. The absorbance from 400 nm to 700 nm was recorded after collecting the supernatant from each time point using a Synergy H1 Hybrid plater reader (BioTek).

To generate a correlation between 620/420 ratios of bromocresol green dye and pH, NaOH was gradually added to the GlsA assay solution, and pH elevation was recorded after each addition. A portion of the solution was also taken out at after each additional spectral scan from 400- to 700-nm wavelengths. The spectrum values were normalized by considering OD values of 0.1 at 515 nm, the isosbestic point (515 nm) of bromocresol green dye.

### ICP-MS analyses to determine cellular manganese levels.

The cells were grown in the presence and absence of manganese for 4 h at 37°C and then pelleted. The pellets were washed twice with 1× PBS supplemented with 1 mM EDTA. The final washing was done with 1× PBS. The washed cell pellets were digested by adding 500 μL of 30% H_2_O_2_ and 3.5 mL of concentrated HNO_3_. The samples were heated over flame for 3 min and made up the final volume of 10 mL with MilliQ water. Digested samples were centrifuged at 10,000 rpm for 20 min. The manganese levels in the supernatants were determined using inductively coupled plasma mass spectrometry (ICP-MS) at Punjab Biotechnology Incubator, Mohali, India. The cellular metal concentrations were calculated by considering a total cellular protein concentration of 300 mg/mL as described (45).

### Statistical analyses.

The experiments were performed 3 to 6 times. Data analyses, normalization, etc. were done using Microsoft Excel, Microcal Origin, and GraphPad Prism software. Densitometry of the Western blots was done using Image J software. The graphs were plotted as mean ± standard deviation (SD). The *P* values were determined from the unpaired *t* test.

### Data availability.

The microarray data generated and used in this study have been deposited in the Gene expression Omnibus (GEO) database with accession number GSE186657.
